# Pharmacogenomics and Opioid Analgesics: Clinical Implications

**DOI:** 10.1155/2015/368979

**Published:** 2015-05-14

**Authors:** Eugenia Yiannakopoulou

**Affiliations:** Department of Medical Laboratories, Faculty of Health and Caring Professions, Technological Educational Institute of Athens, Athens, Greece

## Abstract

Variation exists in patient response on analgesic treatment in terms of efficacy and safety. This variation may be in part explained by pharmacogenomics. This paper aimed to review data on pharmacogenomics of opioid analgesics focusing on the effect of genetic variation on the efficacy and safety of these agents. Current evidence suggests that pharmacogenomics contribute to variation in efficacy and safety of opioids. However, most data come from case control studies and case reports. In addition, a recognized drawback in the field of pharmacogenomics is the common occurrence of false positive association between polymorphisms and the investigated outcome. Prospective studies are needed to further elucidate the clinical implications of available data as well as to define the guidelines for the clinical application of pharmacogenomic data. Furthermore, basic research should focus on the identification of biologically meaningful polymorphisms enabling a hypothesis with biological plausibility driven research in the field of pharmacogenomics of analgesics. Moreover, the publication of relevant negative results should be favoured.

## 1. Introduction

There is great variation in the response of individuals to standard doses of drug therapy that can lead to treatment failure or to life-threatening adverse drug reactions among patients with identical doses of the same drug. A drug's activity is the result of the interaction of the drug with proteins involved in absorption, distribution, metabolism, elimination (ADME proteins), and molecular drug targets or target pathways. Genetic variation in these proteins, that is, single nucleotide polymorphisms in genes coding for metabolizing enzymes or drug transporters, might have a significant influence on the drug effect. Pharmacogenomics investigates interindividual genetic variability in DNA sequence of drug targets, drug metabolizing enzymes or disease genes, RNA expression, or protein translation of genes affecting drug response and drug safety [1].

Although clinical trials including thousands of patients may generally indicate efficacy and lack of toxicity of drugs, it is well known that it is not possible to predict* a priori* whether an individual patient will respond to a given medication without adverse effects. Individual variability in drug response can be explained by a number of parameters including age, gender, weight, pharmacokinetics, disease severity, concomitant diseases, and environmental factors. Moreover, genetic differences among individuals in drug metabolism and/or cellular drug targets may explain a significant component of this variability [2]. In particular interindividual variability in drug metabolism is a major cause of adverse drug effects. In many cases, such variability is linked to polymorphisms in genes coding for drug-metabolizing enzymes. Individuals carrying enzyme-inactivating mutations display impaired drug metabolism. Thus, carriers of inactivating mutations when treated at standard doses have higher plasma drug concentrations and lower clearance rates, rendering them susceptible to adverse drug reactions [3]. There are two types of adverse drug reactions, Type A and Type B. Type A adverse drug reactions are dose-dependent, referring to the augmentation of pharmacological action. Polymorphisms of pharmacokinetic genes may be important for narrow therapeutic window drugs, with poor metabolizers having increased risk of Type A adverse drug reactions. Polymorphisms of drug targets may be also important for this type of adverse drug reactions. Type B idiosyncratic adverse drug reactions are not predicted by the pharmacological action. Idiosyncratic adverse drug reactions are thought to account for up to 20% of all adverse drug reactions, although some researchers regard this as an overestimate, with 5% being closer to reality [4]. Genetic variations of pharmacokinetic genes have been implicated in the aetiology of some idiosyncratic adverse drug reactions.

Narcotic analgesics are widely used for the treatment of severe pain, especially cancer pain. Morphine and other mu-opioid agonists are among the most commonly prescribed narcotic analgesics for moderate to severe pain. There is great variation in human response to opioid analgesia. This variation could be explained by genetic variation in metabolizing enzymes and transporters mediating opioid pharmacokinetics as well as by genetic variation in receptors and signal transduction elements mediating pharmacodynamics. This paper aims to review data on pharmacogenomics of opioid analgesics.

## 2. Pharmacogenomics of Opioid Receptors and Opioid Analgesics

The pharmacologic actions of opioids are mediated through their interaction with the opioid receptors that are G-protein coupled receptors located in the brain and spinal cord. Three subtypes have been cloned, mu-opioid receptors, kappa-opioid receptors, and delta-opioid receptors. Delta receptors are the natural targets of enkephalins. The mu-opioid receptor is the primary site of action of opioid analgesics including morphine, fentanyl, and methadone. Although most of the currently prescribed opioids are mu-opioid receptor agonists, it has been well demonstrated that they exhibit overlapping affinity with kappa- and delta-opioid receptors.

More thoroughly investigated are polymorphisms of mu-opioid receptor gene that have been implicated in variation in opioid response. Data on polymorphisms of kappa- and delta-opioid receptors are quite scarce. More than 100 polymorphisms have been identified in the human mu-opioid peptide receptor gene (*OPRM1*). A well-studied polymorphism of the mu-opioid receptor, the A118G polymorphism, has been described. Depending on the ethnicity, this polymorphism can be found in 2% to 48% of the population. This polymorphism has been associated with both agonistic and antagonistic opioid effects. For example, it has well been demonstrated that the mu-opioid receptor single nucleotide polymorphism (rs number 1799971) at nucleotide position 118 (*OPRM1* 118A>G) resulting in the substitution of one amino acid has been associated with decrease in the analgesic effect of opioids. Data from clinical trials suggest the A118G polymorphism has clinical implication in pain treatment [5]. A cohort study of patients submitted to knee arthroplasty surgery and treated for postoperative pain with patient controlled analgesia was genotyped for the A118G polymorphism in* OPRM1*. The study demonstrated that homozygous GG patients required higher doses of morphine during the first 24 hours [6]. This effect of A118G variant was also verified in a cohort of patients submitted to abdominal surgery [7]. Another study of postoperative pain has shown that patients with the AA genotype need lower doses of morphine but have higher incidence of postoperative nausea and vomit [8]. This finding indicates that* OPRM1* pharmacogenomics might have relevance for opioid side effects. However, there are also studies that demonstrated no effect of A118G polymorphism on opioid induced postoperative nausea and vomit [9].

Studies performed in cancer patients have also indicated the relevance of A118G polymorphism of* OPRM1* in opioid efficacy in pain management [10]. Interestingly, a very recent study has suggested that mu-opioid receptor A118G polymorphism predicts survival in patients with breast cancer [11].

In conclusion, available data indicate that genotyping for mu-opioid receptor A118G polymorphism might have clinical implications in pain management. However, this is a suggestion that needs to be further investigated. Defining* OPRM1* haplotypes might be more clinically relevant as has been suggested by studies of postoperative pain after major abdominal surgery [12]. In addition other common mu-opioid receptor polymorphisms have been associated with reduced pain relief to alfentanil or morphine. In addition, polymorphisms of the mu-opioid receptor have been identified in the third intracellular loop of the receptor that affect receptor signalling, but it is not known if these polymorphisms affect treatment efficacy.

### 2.1. Pharmacogenomics of Phase I Drug Metabolizing Enzymes and Opioid Analgesics

Most Phase I metabolizing enzymes belong to the cytochrome P450 (CYP) family. CYPs (e.g., CYP3A4, CYP3A5, CYP2D6, CYP1A1, CYP1B1, and CYP2E1) recognize a wide range of chemicals as substrates, usually converting them into a more water soluble form. Phase I pathways include oxidative, reductive, and hydrolytic reactions.

#### 2.1.1. Codeine

Codeine is an important P450 2D6 (CYP2D6) substrate. It is activated to morphine exclusively by CYP2D6. Codeine has only mild opioid properties, while most of its analgesia and central nervous system depressant effects are based to its biotransformation to morphine, a reaction catalyzed by CYP2D6 [13]. Genetic polymorphisms of this enzyme result in three phenotypes: poor metabolizer phenotype, extensive metabolizer phenotype, and ultrarapid metabolizer phenotype. Ultrarapid metabolizers have duplication of the gene, resulting in increased enzymatic activity. On the other hand, poor metabolizers are homozygous for an inactive or deficient* CYP2D6* enzyme due to mutations in the* CYP2D6* gene [14]. Poor metabolizers have decreased activation of* CYP2D6*-dependent analgesic prodrugs such as codeine [15]. It is estimated that 7–10% of the population does not express functional CYP2D6. Data from a relevant randomized, placebo-controlled, double-blind clinical trial that investigated the effect of* CYP2D6* polymorphisms on codeine analgesia using an experimental pain model, indicated that codeine administration resulted in analgesia in extensive metabolizers but had no effect in poor metabolizer patients [16]. Furthermore, although poor metabolizers did not receive any analgesic benefit, they had the same frequency of side effects with extensive metabolizers [16]. In a small study of 11 patients treated with codeine for analgesia after hysterectomy, two patients had no analgesic effect from the codeine, one of whom was subsequently shown to be a poor metabolizer [17]. However, other studies have shown no difference in the effect of codeine on postoperative pain intensity between extensive and poor metabolizers in both adults and children [18, 19].

CYP2D6 ultrarapid metabolizers (in an estimated 1% of people in Finland and Denmark, 10% of people in Greece and Portugal, and 29% of people in Ethiopia) have the potential for increased production of morphine from codeine and thus might be at greater risk for opioid related adverse events and might benefit from a lower dose of opioids [20]. The most common adverse reactions to codeine include drowsiness, lightheadedness, dizziness, sedation, shortness of breath, nausea, vomiting, and sweating. Serious adverse reactions include respiratory depression, circulatory depression, respiratory arrest, shock, and cardiac arrest. Excessive activation of codeine in ultrarapid metabolizers with one additional copy of* CYP2D6* has been reported in case reports (Table 1). The first described a patient prescribed a cough medicine containing codeine for bilateral pneumonia, who suffered from a life-threatening opioid intoxication. Upon genotyping, it was shown that the patient had at least three copies of* CYP2D6*, a finding consistent with ultrarapid metabolism of codeine [21]. The patient was also treated with a macrolide and an azole derivative; medicines that, being inhibitors of CYP3A4, might have further reduced the clearance of codeine and increased the risk of an opioid overdose associated with the* CYP2D6* gene duplication. In addition, the patient was suffering from renal insufficiency that rendered him prone to the accumulation of codeine metabolites.

Another case report concerns the death of a two year old boy that developed fever and wheezing and finally respiratory arrest two days after elective tonsillectomy. The boy had been prescribed codeine and acetaminophen for analgesia at recommended doses, that is, 10 to 12.5 mg of codeine and 120 mg of acetaminophen orally every 4 to 6 hours as needed. Codeine and morphine were detected at peripheral blood and* CYP2D6* genotyping revealed functional duplication of the* CYP2D6* allele, resulting in the ultrarapid-metabolizer phenotype [22]. In addition, the child had a history of recurrent episodes of hypoxemia due to bronchopneumonia that could possibly have led to alterations in the *μ*-opioid receptor and increased sensitivity to morphine. Voronov et al. have reported another case report of a 29 month old previously healthy child who experienced apnea resulting in brain injury following a dose of acetaminophen and codeine 2 days after tonsillectomy. A genetic polymorphism leading to ultrarapid metabolism of codeine into morphine was again confirmed in this child [23].

Another case report concerned the death of a breast fed baby 13 days after birth. His mother was prescribed codeine as an analgesic after delivery. Postmortem examination of stored breast milk samples showed morphine levels 4 times higher than expected. Upon genotyping, the mother was found to be heterozygous for a* CYP2D6* ^*∗*^ *2A* allele and a* CYP2D6* ^*∗*^2 × 2 gene duplication. Thus the mother had three functional* CYP2D6* alleles and was classified as an ultrarapid metabolizer [24]. The extra CYP2D6 enzyme resulted in increased* O*-demethylation of codeine to morphine, and consequently, very high concentrations of morphine were found in both the breast milk and in the blood from the child. Following this fatal case, a case control study investigated the characteristics of mothers and infants with or without signs and symptoms of central nervous system depression following codeine exposure during breast feeding. According to the study, breastfed infants of mothers who are CYP2D6 ultrarapid metabolizers combined with the* UGT2B7* ^*∗*^ *2/* ^*∗*^ *2* are at increased risk of potentially life-threatening central nervous system depression [25].

Since 2007, the Food and Drug Administration requires the manufacturers of prescription codeine products to include information in the “Precautions” section of the label to inform prescribing doctors about these risks and to help prevent morphine overdose in breastfed infants.

In a very recent publication, Kelly et al. reported three cases of fatal or life-threatening events associated with codeine. In the 2 fatal cases, functional gene duplications encoding for CYP2D6 caused a significantly greater production of potent morphine from its parent drug, codeine, while a severe case of respiratory depression occurred in an extensive metabolizer [26]. A very recent publication issued guidelines for codeine therapy in the context of* CYP2D6* genotype [27]. According to this publication, alternative analgesics should be prescribed in patients with CYP2D6 poor metabolizer phenotype or ultrarapid metabolizer phenotype. Opioids not metabolized by CYP2D6, including morphine, oxymorphone, buprenorphine, fentanyl, methadone, and hydromorphone combined with nonopioid analgesics, have been proposed as alternative medications instead of codeine for patients prone to codeine toxicity or for those expected to be unresponsive in codeine treatment [27].

#### 2.1.2. Oxycodone

Oxycodone is a semisynthetic opioid prescribed for moderate to severe pain. Oxycodone is similar in structure to codeine, but in contrast, it has an analgesic potency in humans similar to morphine. Both the parent compound and the metabolites of oxycodone are equally active in the opioid receptor. The principal metabolic pathway of oxycodone is the cytochrome P450 3A4 (CYP3A) accounting for approximately 50% of the dose. Furthermore, the drug undergoes metabolism to oxymorphone via CYP2D6. The effect of polymorphisms of CYP enzymes on oxycodone safety and efficacy has been investigated, but results are discordant [28–30].

#### 2.1.3. Hydrocodone

Hydrocodone is hemisynthetic oral opioid structurally related to codeine. It is approximately 12 times more potent in the mu-opioid receptor than codeine. Hydrocodone is metabolized by oxidative metabolism via cytochrome CYP2D6 and cytochrome P450 3A4 (CYP3A4) into hydromorphone and norhydrocodone, respectively. These metabolites undergo conjugation by uridine diphosphate glucuronosyltransferases (UGTs) before excretion. Both the parent compounds and the metabolites of hydrocodone are equally active in opioid receptors. An effect of CYP2D6 polymorphism on hydrocodone metabolite production has been demonstrated. However, evidence suggests that analgesic efficacy of hydrocodone is not affected by metabolism via CYP2D6. Effect of CYP2D6 polymorphisms on hydrocodone toxicity has also been reported (Table 2). Madadi et al. reported a case of fatal opioid toxicity that occurred in a developmentally delayed child aged 5 years 9 months who was inadvertently administered high doses of hydrocodone for a respiratory tract infection. Genotyping was performed for* CYP2D6* ^*∗*^ *4*,* CYP2D6* ^*∗*^ *9*,* CYP2D6* ^*∗*^ *10*, and* CYP2D6* ^*∗*^ *41* by using predesigned TaqMan allelic discrimination assays. Furthermore, the following assays were applied: (1)* CYP2D6* copy number attributable to the* CYP2D6* ^*∗*^ *5* deletion allele (2) gene duplication (3) gene multiduplication. The analysis demonstrated that the child carried the allele* CYP2D6* ^*∗*^ *41* allele and 1 fully functional wild-type allele. Therefore, the child genotype was* CYP2D6* ^*∗*^ *2A/* ^*∗*^ *41* and the child was designated as a poor CYP2D6 metabolizer. Drug-drug interactions contributed to this fatal event, since the child was prescribed clarithromycin (a CYP3A4 inhibitor) for an ear infection and valproic acid, an inhibitor of UGT enzymes for seizures, since birth [31, 32].

#### 2.1.4. Tramadol

Tramadol is a synthetic, centrally acting analgesic for the treatment of moderate to severe pain. This analgesic is a racemic mixture containing 50% (+)tramadol and 50% (−)tramadol. Tramadol produces analgesia by the synergistic action of its two enantiomers and their metabolites. Its affinity for opioid receptors is 6000 times lower than that of morphine. Tramadol is an example of analgesic drug that is metabolized by CYP2D6, to generate a pharmacologically active product, the analgesic opioid receptor agonist O-desmethyltramadol [33]. Genetic variations in* CYP2D6* have been shown to account for some of the variable pain response in the postoperative period because the* CYP2D6* activity has a clinically relevant impact on the level of analgesia mediated by the *μ*-opioid receptor [34–36] (Table 3). The effect of* CYP2D6* ^*∗*^ *10* polymorphism has been investigated in Chinese patients recovering from gastrectomy performed for gastric cancer [37]. Patients were treated with self-administered tramadol via patient controlled analgesia. Patients homozygous for* CYP2D6* ^*∗*^ *10* displayed higher consumption of tramadol in comparison with heterozygous group and with patient group without* CYP2D6* ^*∗*^ *10*. Furthermore, tramadol is metabolized to N-demethyl tramadol by N-demethylation via CYP3A.

In addition genetic variations in CYP2D6 affect the safety profile of tramadol. Thus, case reports have indicated that the carriers of gene duplications, being CYP2D6 ultrarapid metabolizers, are at high risk for toxic responses to tramadol treatment [38]. A case report described a man with renal insufficiency and CYP2D6 UM genotype who developed postoperative opioid related respiratory insufficiency under intravenous patient controlled tramadol analgesia [39]. Genotyping was performed by polymerase chain reaction and real-time polymerase chain reaction for the single nucleotide polymorphisms* CYP2D6* ^*∗*^ *3,* ^∗^ *4,* ^∗^ *5,* ^∗^ *6,* ^∗^ *7, *and ^∗^ *8*, associated with poor metabolizer phenotype and for the SNP* CYP2D6* ^*∗*^ *10* and ^∗^ *41* associated with the intermediate-metabolizer phenotype. The result of these tests was negative, while the duplication/multiduplication assay revealed a CYP2D6 gene duplication resulting in ultrarapid metabolism of tramadol to its active metabolite (+)*O*-desmethyltramadol. The opioid toxicity was enhanced by the decreased metabolic clearance due to renal insufficiency.

In another publication, Elkalioubie et al. have reported a near fatal case of tramadol induced cardiotoxicity in an ultrarapid CYP2D6 metabolizer [40]. A 22-year-old girl was transferred to the ICU with cardiac arrest. The aetiology was suggested by the high concentrations of both tramadol and its main metabolite O-desmethyltramadol identified in the blood tests. Genotyping was performed and the patient was found to be heterozygous for a wild-type allele duplication. The patient admitted occasional abuse of tramadol. Cardiotoxicity could be explained by high levels of norepinephrine due to tramadol induced inhibition of norepinephrine reuptake.

Motivated by the above mentioned reports, Kirchheiner et al. systematically investigated the effect of* CYP2D6* duplication on the pharmacodynamics and pharmacokinetics of tramadol after a single dose of 100 mg racemic tramadol in 11 carriers of a* CYP2D6* gene duplication and compared with 11 carriers of 2 active* CYP2D6* genes [38]. In addition, Siew et al. investigated the effect of* CYP2D6* polymorphisms on tramadol pharmacokinetics and pharmacodynamics in Malaysian patients with different genotypes [35]. Both studies demonstrated pharmacokinetic differences between the genotype groups as well as differences in the safety profile of tramadol. According to Gal et al. intermediate metabolizers experienced more adverse effects than extensive metabolizers and extensive metabolizers experienced more adverse effects than ultrarapid metabolizers. However, in the study of Kirchheiner et al., ultrarapid metabolizers experienced more adverse events than extensive metabolizers. The discrepancies may be due to differences in the method of genotype determination, in the different polymorphisms investigated, and in the type of assessed adverse events. Given that these results have not been verified in a large population study, at present,* CYP2D6* genotyping cannot be recommended for tramadol dosing.

#### 2.1.5. Methadone

Methadone metabolism is attributed primarily to cytochrome P450 enzymes CYP3A4, CYP2B6, and CYP2D6 [41, 42]. Interindividual differences in sensitivity to methadone have been observed. Fatal poisonings occur typically at concentrations between 0.4 and 1.8 mg/mL. However, in susceptible individuals, death may occur at much lower concentrations. In a study, 40 postmortem cases have been reported, in which methadone had been implicated in the cause of death. The* CYP2B6* ^*∗*^ *6* allele that results in slow metabolism was associated with highest postmortem methadone concentration [43].

### 2.2. Pharmacogenomics of Phase II Drug Metabolizing Enzymes and Opioid Analgesics

Phase II enzymes conjugate phase I metabolites, other intermediates, or the parent compound for renal or biliary excretion. Phase II enzymes include glutathione S transferases (GSTs), UDP glycosyltransferases (UGT), N-acetyltransferases (NAT), glutathione S transferases GSTs, NADH quinone oxidases, and others. Knowledge on pharmacogenomics of phase II enzymes is not extensive. Thus, the relevance of the pharmacogenomics of the enzymes of the uridine diphosphoglucuronosyltransferase (UGT) superfamily in opioid disposition has been investigated, although it is not currently elucidated [44–47]. Several polymorphisms of* UGT1A1* have been described with the best studied the one defined by a variable length “TA” tandem repeat in the regulatory TATA box of the* UGT1A1* gene promoter (*UGT1A1* ^*∗*^ *28*) that leads to reduced expression of the isozyme, associated with Gilbert syndrome, the most common inherited cause of unconjugated hyperbilirubinemia. UGT1A1 catalyzes the glucuronidation of opioids including morphine, buprenorphine, and norbuprenorphine, hemisynthetic derivatives of the morphine alkaloid thebaine with partial agonist properties on opioid receptors. The effect of* UGT1A1* ^*∗*^ *28* polymorphism in the pharmacokinetics of morphine has been investigated in cancer patients, but no association has been demonstrated [45]. Another enzyme involved in opioid metabolism is UGT2B7. The association of polymorphisms of this enzyme with morphine metabolism has been investigated. It has been shown that the* UGT2B7* promoter variant −840 G is associated with reduced glucuronidation of morphine contributing thus in variability in hepatic clearance of morphine in patients with sickle cell anaemia [46]. Genetic modeling approaches integrating prior physiologic knowledge have shown that decreased UGT2B7 activity is associated with a decrease in active opioid exposure. The C802T exonic single nucleotide polymorphism (SNP) of* UGT2B7* has been shown to alter UGT2B7 affinity for buprenorphine.

## 3. Pharmacogenomics of Drug Transporters and Opioid Analgesics

Transport of drugs across cell membranes is determined by molecular weight, lipid solubility, ionization, and protein binding. Opioid analgesics are hydrophilic agents and thus the drug transporters mediate their entrance through cell membranes. Multidrug resistance proteins (MDR), multidrug resistance associated proteins (MRP), and organic anion transporting polypeptides (OATPs) contribute to opioid analgesic transport through cell membranes. Polymorphisms in these transporter genes may account for variation in analgesic efficacy of opioids. MDRs are ATP dependent efflux pumps. P glycoprotein is encoded by the multidrug resistance gene* MDR1* (*ABCB1*). The clinical relevance of* ABCB1* pharmacogenomics in the case of opioids has not been elucidated. Evidence suggests that the variations in the P-glycoprotein gene,* ABCB1* polymorphisms, influence opioid pharmacodynamics and dosage requirements. However, there are also studies that failed to demonstrate an effect of* ABCB1* polymorphisms on morphine pharmacodynamics or pharmacokinetics. Fentanyl may be a substrate of ABCB1 and it has been shown that* ABCB1* polymorphisms conferring decreased transporter function have been associated with increased respiratory depressive effects of fentanyl in Korean patients [48]. A cohort study of patients submitted to colorectal surgery demonstrated that two SNPs of ABCB1, C3435T (in exon 24) and G2677T/A (in exon 21), were associated with opioid induced side effects. In particular, homozygous patients (GG and CC, resp.) required less often ondansetron treatment, suggesting that the diplotype GG-CC was associated with fewer side effects of morphine [49]. However, a recent study in Turkish patients failed to demonstrate an effect of* ABCB1* polymorphisms on respiratory depression caused by intravenous fentanyl [50].

Further research is needed in order to elucidate the clinical relevance of* MRP* and* OATP* polymorphisms in the context of opioid analgesics efficacy and safety.

## 4. Conclusion

Undoubtedly, the application of personalized medicine is anticipated to improve treatment efficacy and safety. Pharmacogenomics may be a critical pathway to personalized medicine. Current evidence suggests that pharmacogenomics contribute to variation in efficacy and safety of opioids. However, most data come from case control studies and case reports. In addition, a recognized drawback in the field of pharmacogenomics is the common occurrence of false positive association between polymorphisms and the investigated outcome. Prospective studies are needed to further elucidate the clinical implications of available data as well as to define the guidelines for the clinical application of pharmacogenomic data. Furthermore, basic research should focus on the identification of biologically meaningful polymorphisms enabling a hypothesis with biological plausibility driven research in the field of pharmacogenomics of analgesics. Moreover, the publication of relevant negative results should be favored.

## Figures and Tables

**Table 1:** Case reports providing evidence on the impact of polymorphisms of metabolizing enzymes on the safety of codeine.

Author	Metabolizing enzyme	Polymorphism	Adverse event
Gasche et al., 2004 [21]	CYP2D6	CYP2D6^∗^1 × 3, in a patient suffering from renal insufficiency and cotreated with CYP3A4 inhibitors	Life-threatening intoxication
Voronov et al., 2007 [23]	CYP2D6	CYP2D6 × 2	Apnea and brain injury
Madadi et al., 2007 [24]	CYP2D6	CYP2D6^∗^2A and CYP2D6^∗^2 × 2	Death of the breastfed 13-day-old boy
Ciszkowski et al., 2009 [22]	CYP2D6	CYP2D6^∗^1 × N	Death due to respiratory arrest
Kelly et al., 2012 [26]	CYP2D6	CYP2D6^∗^1 × N	Two deaths, one case of severe respiratory depression

**Table 2:** Case reports providing evidence on the impact of polymorphisms of metabolizing enzymes on the safety of hydrocodone or oxycodone.

Author	Metabolizing enzyme	Polymorphism	Adverse event
Madadi et al., 2010 [32]	CYP2D6	CYP2D6^∗^41	Death

Samer et al., 2010 [28]	CYP2D6	32 alleles using the AmpliChip TMCYP450 DNA microarray	Volunteers who were CYP2D6 ultrarapid metabolizers experienced higher toxicity especially after CYP3A blockade with ketoconazole

Lemberg et al., 2010 [29]	CYP2D6, CYP3A4, CYP3A5		No effect of genotype in patients treated for malignant and nonmalignant chronic pain

Andreassen et al., 2012 [30]	CYP2D6	CYP2D6^∗^2 × 2	Genotype did not affect safety profile that is the incidence of nausea, tiredness, or cognitive failure in a cohort of cancer patients

**Table 3:** Prospective studies providing evidence on the impact of polymorphisms of CYP2D6 on the analgesic efficacy of tramadol.

Author	Polymorphism	Effect
Stamer et al., 2003 [34]	CYP2D6^∗^1, ^∗^3, ^∗^4, ^∗^5, ^∗^9, ^∗^10, CYP2D6^∗^17, CYP2D6^∗^1 × N	Poor metabolizers for CYP2D6 showed a lower response rate to tramadol analgesia for postoperative pain after abdominal surgery

Wang et al., 2006 [37]	CYP2D6^∗^10 C188T	This SNP reduced tramadol analgesic efficacy in a Chinese patients treated for postoperative pain after major abdominal surgery

Siew et al., 2007 [35]	CYP2D6^∗^1, ^∗^3, ^∗^4, ^∗^5, ^∗^9, ^∗^10, CYP2D6^∗^17, CYP2D6^∗^1 × N	The analgesic effects of tramadol were not measured adequately. Therefore the effect of genotype on tramadol analgesic efficacy was not demonstrated

Slanar et al., 2012 [36]	CYP2D6^∗^3, CYP2D6^∗^4, CYP2D6^∗^5, CYP2D6^∗^6 CYP2D6^∗^1 × N	CYP2D6 genotype did not affect tramadol analgesic efficacy in postoperative patients after knee arthroplasty
